# Metabolic-Associated Steatotic Liver Disease and FGF21 Dysregulation in Seipin-Deficient and *BSCL2*-Associated Celia’s Encephalopathy Murine Models

**DOI:** 10.3390/ijms262412037

**Published:** 2025-12-14

**Authors:** Silvia Cobelo-Gómez, Lía García-Formoso, Antía Fernández-Pombo, Héctor Lázare-Iglesias, Everardo Díaz-López, Teresa Prado-Moraña, Laura Rodríguez-Sobrino, Ana Senra, David Araújo-Vilar, Sofía Sánchez-Iglesias

**Affiliations:** 1 UETeM-Molecular Pathology Group, Department of Psychiatry, Radiology, Public Health, Nursing and Medicine, Instituto de Investigación Sanitaria Santiago de Compostela—Center for Research in Molecular Medicine and Chronic Diseases (IDIS-CIMUS), University of Santiago de Compostela, 15782 Santiago de Compostela, Spain; silviacobelog@gmail.com (S.C.-G.); lia.garcia.formoso@sergas.es (L.G.-F.); antia.fernandez@usc.es (A.F.-P.); everardojosue.diaz@rai.usc.es (E.D.-L.); teresa.prado@rai.usc.es (T.P.-M.); laurarodriguez.sobrino@usc.es (L.R.-S.); 2 Department of Pathology, University Clinical Hospital of Santiago de Compostela, 15706 Santiago de Compostela, Spain; hector.lazare.iglesias@sergas.es; 3 CIMUS Biomedical Research Institute, University of Santiago de Compostela-IDIS, 15782 Santiago de Compostela, Spain; ana.senra@usc.es

**Keywords:** CGL2, PELD, liver, MASLD, Fgf21, BSCL2, seipin

## Abstract

Seipin, a protein encoded by the *BSCL2* gene, plays a crucial role in lipid metabolism, and some pathogenic biallelic variants cause lipodystrophy and associated metabolic disorders. This study investigates liver pathology and dysregulation of the *FGF21* signalling pathway in two mouse models: *Bscl2*^−/−^ (knock-out) and *Bscl2*^Celia/Celia^ (knock-in). We evaluated liver histopathology using H&E and Oil red O staining, assessed hepatic triglyceride levels via enzymatic assays, and analyzed gene expression of key *FGF21*-related components (*Fgf21*, *Ppargc1a*, *Fgfr1*, and *Klb*) using quantitative real-time PCR. The liver histology was scored using the NAFLD activity score (NAS) system. Both models exhibited hepatic steatosis and inflammatory features. The *Bscl2*^−/−^ mice showed more pronounced liver damage, including ballooning degeneration and fibrosis. Gene expression analysis revealed a significant increase in *Fgf21* in both models, suggesting an adaptive response to liver injury. Notably, *Fgfr1* and *Ppargc1a* expression was moderately elevated in severe neurologically affected mice showing less hepatic involvement, suggesting a potential adaptive or protective association of these genes with reduced steatosis. Seipin deficiency leads to metabolic-associated steatotic liver disease and dysregulated *FGF21* signalling. These findings provide insight into the pathophysiological mechanisms of lipodystrophy and liver disease and suggest that the *FGF21* pathway could be a therapeutic target for treating seipin-related metabolic disorders.

## 1. Introduction

Congenital generalized lipodystrophy type 2 (CGL2) is a very rare autosomal recessive disorder caused by pathogenic variants in the *BSCL2* gene. Patients usually present with a generalized absence of adipose tissue that is evident from birth or during the first year of life [1]. These children have early hyperinsulinemia, hypoleptinemia, hypoadiponectinemia, and hypertriglyceridemia. In 2013, our group described a new neurodegenerative disease in children, Celia’s encephalopathy or progressive encephalopathy with or without lipodystrophy (PELD) [2] (MIM: #615924). This disease could be considered a specific variant of CGL2 with a devastating neurological picture. This extremely rare disease is due to different variants in the *BSCL2* gene. The most common and first described was the variant c.985C>T, in exon 7, in homozygosity or compound heterozygosity. Homozygous patients may not show typical features of generalized lipodystrophy, while compound heterozygotes often show signs of CGL2 in early childhood.

These diseases represent an extreme model of ectopic fat accumulation, typically associated with the development of an exaggerated metabolic syndrome [3]. In these patients, reduced subcutaneous adipose tissue combined with leptin deficiency makes the liver the main store of triglycerides [3]. Leptin levels are directly proportional to adipose tissue mass [4], with hypoleptinemia characteristic of lipodystrophies and hyperleptinemia characteristic of obesity. In both situations, hepatic steatosis is associated with a decrease in leptin action [3] (either due to deficiency in the former case or resistance in the latter) and an increase in the flux of free fatty acids (FFA) to the liver (either due to the absence of sufficient adipose tissue for normal fat deposition or due to its expansion).

Clinically, liver involvement in congenital generalized lipodystrophy presents as hepatomegaly and often hypertransaminasemia [5]. In these patients, NAFLD, currently renamed metabolic dysfunction-associated steatotic liver disease (MASLD) [6], often fulfils the histological criteria for steatohepatitis, either borderline or definite [7]. It manifests as typical macrovesicular steatosis, zone 3 hepatocellular damage (with ballooning degeneration and Mallory–Denk bodies), predominantly lobular inflammation, and fibrosis [3,7]. Cirrhosis, liver failure, and hepatocellular carcinoma are potential outcomes of metabolic dysfunction-associated steatohepatitis (MASH) progression in these patients [4]. Notably, patients with CGL2 may develop extreme fibrosis (in the form of fibrosis bridging or already established cirrhosis) at an earlier age than patients with other forms of lipodystrophy [7].

In seipin knock-out mice (SKO), both reduced adipogenesis and increased lipolysis lead to a reduction in the number of adipocytes available for triglyceride storage and adipokine secretion [8]. This leads to ectopic lipid deposition, although it cannot be excluded that local loss of seipin in the liver may worsen the metabolic state in the presence of adipose tissue loss or dysfunction [9]. The development of hepatic steatosis in SKO mice has been attributed, at least in part, to increased hepatic lipoprotein uptake [10] and de novo lipogenesis, as well as activation of an alternative triglyceride synthesis pathway mediated by *Mgat1* expression and decreased fatty acid oxidation. In these typically hyperphagic SKO mice, these mechanisms are enhanced by substrate excess [11].

The role of seipin in the liver is controversial. On the one hand, in vitro studies suggest that seipin deficiency in hepatocytes plays an autonomous role in the development of MASLD [12]. On the other hand, there are animal models for [13,14] and against this hypothesis [9,11,15,16]. Thus, the pathogenesis of MASLD in the context of GCL2 or PELD is not fully understood, and several explanations have been proposed to explain the link between seipin deficiency and ectopic lipid accumulation.

Fibroblast growth factor 21 (FGF21) is an endocrine regulator with key roles in lipid and glucose metabolism, mitochondrial function, and adaptive responses to metabolic stress. It is mainly secreted by the liver in response to fasting or lipotoxic stimuli and exerts its biological activity through binding to FGF receptors (primarily FGFR1) in the presence of the co-receptor β-Klotho (KLB). In metabolic-associated steatotic liver disease, elevated circulating and hepatic FGF21 levels have been interpreted as a compensatory response to hepatocellular injury. Given that FGF21 dysregulation has been described in lipodystrophic patients and in seipin-deficient mice [17,18,19], we aimed to characterize the hepatic FGF21 pathway in our models to better understand its potential role in the pathogenesis of seipin-related liver disease.

To date, most of the scientific literature on MASLD in SKO mice has focused on the pathophysiological mechanisms involved in the metabolic alterations and the changes induced by the therapies under investigation. References to the pathological anatomy of the liver are scarce, and long-term histological evolution has never been studied or assessed by a scoring system. Therefore, the present study details liver involvement in a murine model of CGL2 and in a unique model of Celia’s encephalopathy.

## 2. Results

### 2.1. Macroscropic Examination of the Liver

**Figure 1:**
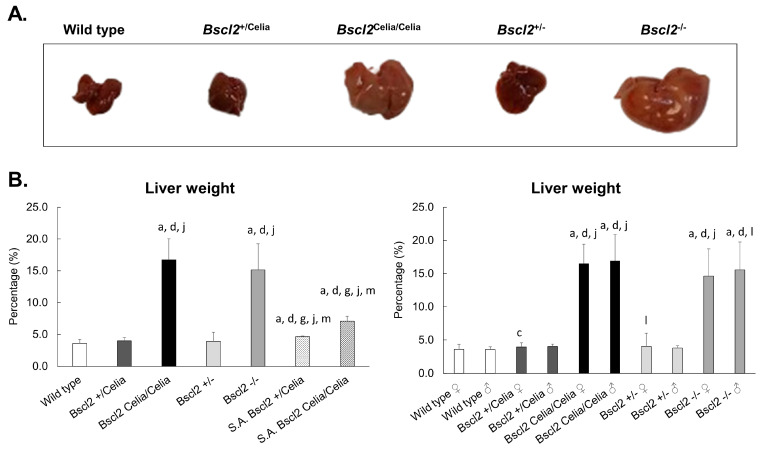
Analysis of the liver tissue. (**A**) Liver macroscopic appearances. 7.2-month-old *Bscl2*^+/−^and *Bscl2*^−/−^; 6.8-month-old wild-type, 6.8-month-old *Bscl2*^+/Celia^ and 5.3-month-old *Bscl2*^Celia/Celia^ ♂ mice. (**B**) Liver weight comparison in terms of percentage. Left panel: all studied animals. Right panel: sex-dependent analysis (males and females shown separately for clarity). For mean age and number of animals per genotype, see Supplementary Table S1. Data is presented as n (%) ± SD. ^a^ *p* < 0.05 vs. wild-type ♀/♂; ^c^ *p* < 0.05 vs. wild-type ♂; ^d^ *p* < 0.05 vs. *Bscl2*^+/−^ ♀/♂; ^g^ *p* < 0.05 vs. *Bscl2*^−/−^ ♀/♂; ^j^ *p* < 0.05 vs. *Bscl2*^+/Celia^ ♀/♂; ^l^ *p* < 0.05 vs. *Bscl2*^+/Celia^ ♂; ^m^ *p* < 0.05 vs. *Bscl2*^Celia/Celia^ ♀/♂. Part of the data used in this study were previously published in [20].

The livers of wild-type and heterozygous mice, *Bscl2*^+/Celia^ and *Bscl2*^+/−^, appeared normal in terms of colour and size. In contrast, homozygous mice, *Bscl2*^Celia/Celia^ and *Bscl2*^−/−^ presented with an enlarged liver and hepatic pallor (Figure 1A), in some cases accounting for up to 25% of the animal’s body weight, which was significantly greater compared to wild-type, heterozygous, and severely affected animals (Figure 1B). The severely affected mice (S.A.) did not exhibit liver pallor, but they did show a greater liver weight compared to wild-type and heterozygous animals. However, no significant differences were found between the S.A. *Bscl2*^Celia/Celia^ and *Bscl2*^+/Celia^ animals, although the S.A. *Bscl2*^Celia/Celia^ mice appeared to show a higher liver weight compared to the S.A. *Bscl2*^+/Celia^ mice (Figure 1B). Furthermore, these S.A. *Bscl2*^Celia/Celia^ mice had much smaller liver sizes compared to the non-S.A. homozygous animals (*Bscl2*^Celia/Celia^ and *Bscl2*^−/−^), more closely resembling the liver characteristics of non-S.A. heterozygous (*Bscl2*^+/Celia^ and *Bscl2*^+/−^) or wild-type mice. No differences in liver characteristics were found between sexes or between the knock-in and knock-out mouse models.

### 2.2. Histopathological Description of the Liver

**Figure 2:**
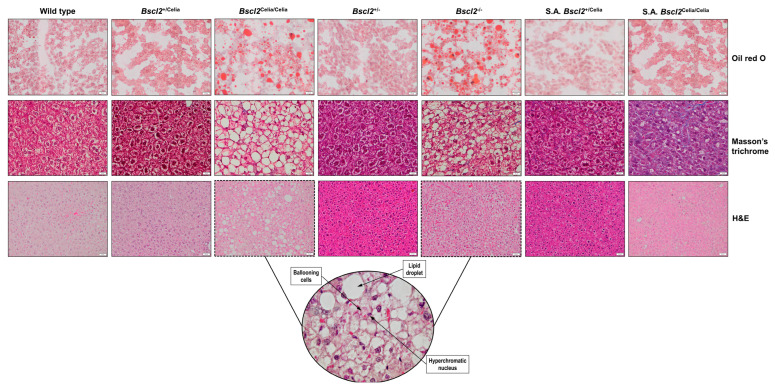
Histology of liver tissue. Oil red O, scale bar: 20 µm, mean age 6 months (wild-type ♀, *Bscl2*^+/Celia^ ♂, *Bscl2*^Celia/Celia^ ♂, *Bscl2*^+/−^ ♂, *Bscl2*^−/−^ ♂) except for S.A. *Bscl2*^+/Celia^ ♂ (8.6-month-old) and S.A. *Bscl2*^Celia/Celia^ ♂ (4-month-old) mice. Masson’s trichrome, scale bar: 10 µm, mean age 9 months (wild-type ♂, *Bscl2*^+/Celia^ ♂, *Bscl2*^Celia/Celia^ ♂, *Bscl2*^−/−^ ♂ and S.A. *Bscl2*^Celia/Celia^ ♂) except for *Bscl2*^+/−^ ♂ (6.1-month-old) and S.A. *Bscl2*^+/Celia^ ♂ (5.8-month-old) mice. H&E, scale bar: 20 µm and 10 µm (round shaped figure), wild-type ♂, *Bscl2*^+/Celia^ ♂ and *Bscl2*^−/−^ ♀ (12-month-old), *Bscl2*^Celia/Celia^ ♀ (14-month-old), *Bscl2*^+/−^ ♂ (6.1-month-old) and severely affected (S.A.) mice: *Bscl2*^+/Celia^ ♂ (5.8-month-old), *Bscl2*^Celia/Celia^ ♂ (19-month-old).

The liver histology of wild-type and heterozygous mice, *Bscl2*^+/Celia^ and *Bscl2*^+/−^, was found to be normal. While the presence of minimal lobular infiltrates was observed in some cases, this was considered a non-specific finding that, in the absence of steatosis and ballooning degeneration, had no diagnostic implications (Figure 2).

**Figure 3:**
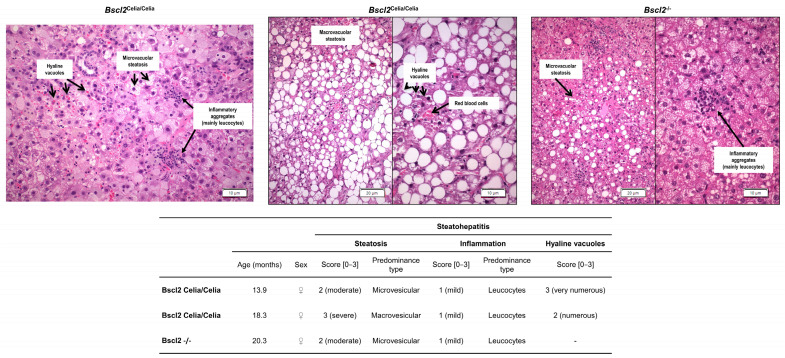
Assessment of steatohepatitis in the liver of homozygous mice. Histological assessment of steatosis, inflammation, and hyaline vacuoles. *Bscl2*^Celia/Celia^ (n = 2) and *Bscl2*^−/−^ (n = 1). H&E.

In contrast, the liver histology of homozygous mice, *Bscl2*^Celia/Celia^ and *Bscl2*^−/−^, revealed the presence of hepatic steatosis as early as 3 months of age, which persisted as a consistent finding from 6 months onwards (Figure 2). This ectopic fat deposition (as shown by Oil red O staining in Figure 2) exhibited a mixed pathological pattern, comprising both macrovesicular steatosis, characterized by large lipid vacuoles that displaced the nucleus to the periphery, and microvesicular steatosis, characterized by small lipid droplets filling the cytoplasm without nuclear displacement (Figure 2 and Figure 3). However, the steatosis was predominantly macrovesicular, accompanied by the presence of ballooning cells with pale, feathery cytoplasms and small, hyperchromatic nuclei (as shown by H&E 10 µm staining in Figure 2).

Foci of lobular inflammation, composed predominantly of mononuclear cells, were also observed with some frequency (Figure 3). The histopathological analysis of *Bscl2*^Celia/Celia^ mice further revealed the presence of intracytoplasmic hyaline bodies (IHBs), round or oval structures with a homogeneous, eosinophilic content, and a surrounding halo, indicative of cellular damage (Figure 3). These IHBs were not detected in the *Bscl2*^−/−^ mice. Additionally, some cases showed a striking chronic perivascular infiltrate.

**Figure 4:**
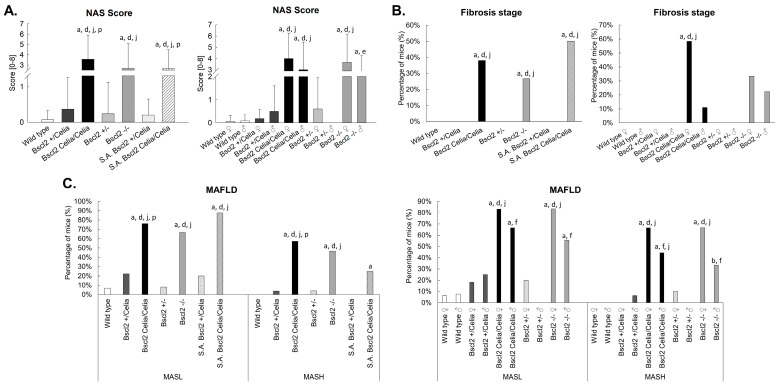
Assessment of liver involvement. (**A**) NAS Score. Degree of activity of non-alcoholic fatty liver disease calculated as the sum of scores for steatosis, ballooning degeneration, and lobular inflammation according to the NAS scoring system of the NASH CRN. Data is presented as mean ± SD. (**B**) Fibrosis stage. Percentage of animals with fibrosis stage 1a in the liver according to the NASH CRN System. (**C**) Evaluation of subtypes of metabolic dysfunction-associated steatotic liver disease (MASLD). Percentage of animals presenting metabolic dysfunction-associated steatotic liver (MASL) and/or metabolic dysfunction-associated steatohepatitis (MASH). For mean age and number of animals per genotype, see Supplementary Table S1. ^a^ *p* < 0.05 vs. wild-type ♀/♂; ^b^ *p* < 0.05 vs. wild-type ♀; ^d^ *p* < 0.05 vs. *Bscl2*^+/−^ ♀/♂; ^e^ *p* < 0.05 vs. *Bscl2*^+/−^ ♀; ^f^ *p* < 0.05 vs. *Bscl2*^+/−^ ♂; ^j^ *p* < 0.05 vs. *Bscl2*^+/Celia^ ♀/♂; ^p^ *p* < 0.05 vs. S.A. *Bscl2*^+/Celia^ ♀/♂. Part of the data used in this study were previously published in [20].

These histological characteristics were collectively grouped and evaluated using the NAFLD activity score (NAS) system to assess MASLD. Following these criteria, homozygous animals, *Bscl2*^Celia/Celia^ and *Bscl2*^−/−^, demonstrated a significantly higher total score for MASLD compared to wild-type and heterozygous mice, *Bscl2*^+/Celia^ and *Bscl2*^+/−^ (Figure 4A). Specifically, the percentage of homozygous animals presenting both MASL (67–76%) and MASH (47–57%) was higher compared to wild-type and heterozygous mice (Figure 4C). Notably, the *Bscl2*^Celia/Celia^ mice had a higher NAS score, and the percentage of animals with MASL and MASH was also higher compared to S.A. *Bscl2*^+/Celia^ mice, a finding not replicated in *Bscl2*^−/−^ mice. Moreover, fibrosis status analysis indicated that 27–38% of homozygous mice with marked active liver damage also developed mild pericellular fibrosis (as shown by Masson’s trichrome staining in Figure 2 and Figure 4B). No differences were observed between sexes, although in the case of homozygous *Bscl2*^−/−^ and *Bscl2*^Celia/Celia^ mice, females tended to have slightly higher hepatic involvement than males.

Furthermore, the S.A. *Bscl2*^Celia/Celia^ mice presented mild hepatic steatosis (Figure 2). Similarly to the non-S.A. *Bscl2*^Celia/Celia^ mice, the 50% of S.A. *Bscl2*^Celia/Celia^ mice exhibited mild pericellular fibrosis (Figure 4B) with a higher total NAS score (Figure 4A) compared to wild-type, heterozygous (*Bscl2*^+/Celia^ and *Bscl2*^+/−^), and S.A. *Bscl2*^+/Celia^ mice. Additionally, 88% of S.A. *Bscl2*^Celia/Celia^ mice showed MASL, of which 25% developed MASH, whereas only 20% of S.A. *Bscl2*^+/Celia^ mice showed signs of MASL, never progressing to steatohepatitis (Figure 4C).

**Figure 5:**
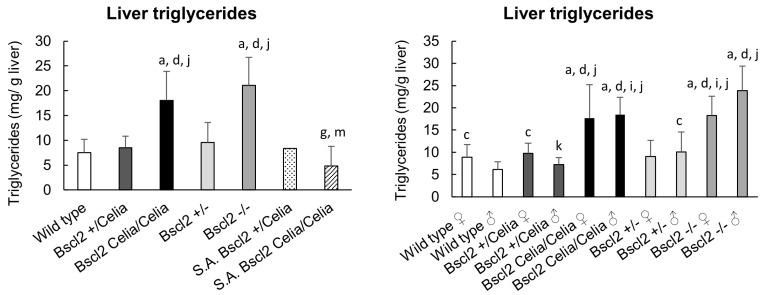
Liver triglycerides concentration for wild-type, *Bscl2*^+/Celia^, *Bscl2*^Celia/Celia^, *Bscl2*^+/−^, *Bscl2*^−/−^, and severely affected (S.A.) animals. Left panel: all animals. Right panel: sex-dependent analysis (male and female data shown separately). Wild-type, *Bscl2*^+/Celia^, *Bscl2*^Celia/Celia^, *Bscl2*^+/−^, n = 20 (10 ♀ and 10 ♂); S.A *Bscl2*^+/Celia^, n = 1 and S.A *Bscl2*^Celia/Celia^, n = 2 animals. Mean age: 9.5 months old. Data is presented as mean ± SD (see Supplementary Table S2). ^a^ *p* < 0.05 vs. wild-type ♀/♂; ^c^ *p* < 0.05 vs. wild-type ♂; ^d^ *p* < 0.05 vs. *Bscl2*^+/−^ ♀/♂; ^g^ *p* < 0.05 vs. *Bscl2*^−/−^ ♀/♂; ^i^ *p* < 0.05 vs. *Bscl2*^−/−^ ♂; ^j^ *p* < 0.05 vs. *Bscl2*^+/Celia^ ♀/♂; ^k^ *p* < 0.05 vs. *Bscl2*^+/Celia^ ♀; ^m^ *p* < 0.05 vs. *Bscl2*^Celia/Celia^ ♀/♂.

The analysis of triglyceride concentration in the liver of the homozygous animals, *Bscl2*^Celia/Celia^ and *Bscl2*^−/−^, showed a significant accumulation of triglycerides (Figure 5). This finding is consistent with the previously described presence of a fatty liver and a marked hepatic steatosis in these animals. In contrast, despite the presence of mild steatosis in the severely affected mice, the concentration of triglycerides in their liver was similar to that of wild-type and heterozygous mice. No obvious differences were found between males and females. However, a higher concentration of triglycerides was observed in the liver of male *Bscl2*^−/−^ mice compared to male *Bscl2*^Celia/Celia^ mice (Figure 5).

### 2.3. Gene Expression in Hepatic Tissue

**Figure 6:**
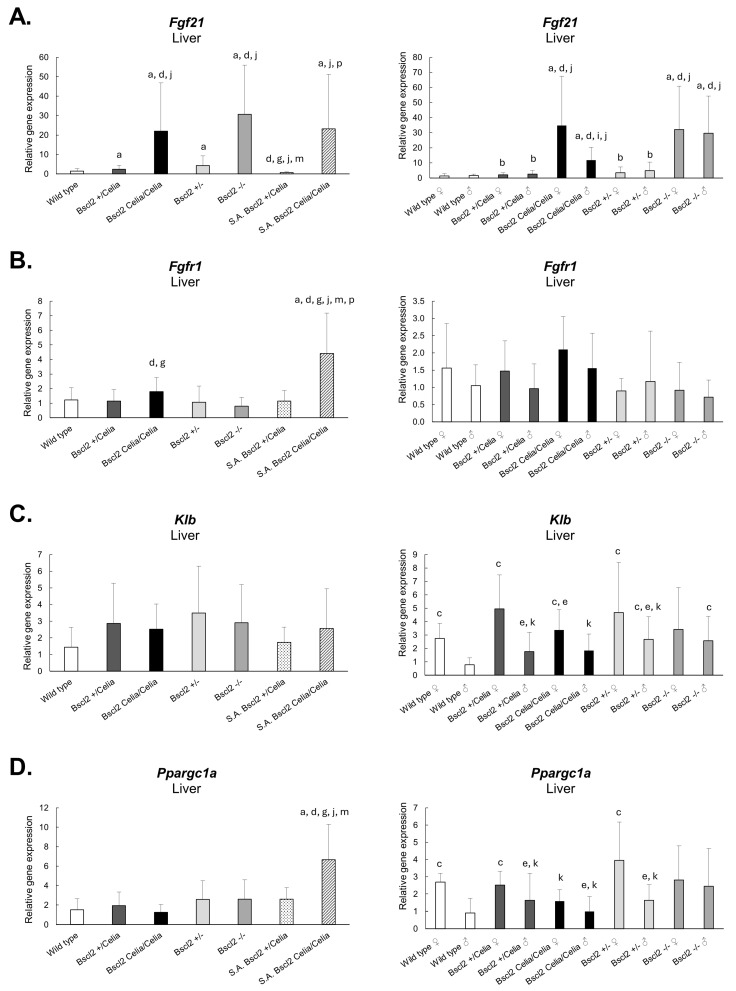
Relative expression of (**A**) *Fgf21,* (**B**) *Fgfr1,* (**C**) *Klb*, and (**D**) *Ppargc1a* genes in liver. Wild-type, *Bscl2*^+/Celia^, *Bscl2*^Celia/Celia^, *Bscl2*^+/−^, *Bscl2*^−/−^, and severely affected (S.A) *Bscl2*^Celia/Celia^ and *Bscl2*^+/Celia^ animals. For mean age and number of animals per genotype, see Supplementary Table S3. Data is presented as mean ± SD. Results were normalized for the *Rn18S* gene and referred to wild-type. ^a^ *p* < 0.05 vs. wild-type ♀/♂; ^b^ *p* < 0.05 vs. wild-type ♀; ^c^ *p* < 0.05 vs. wild-type ♂; ^d^ *p* < 0.05 vs. *Bscl2*^+/−^ ♀/♂; ^e^ *p* < 0.05 vs. *Bscl2*^+/−^ ♀; ^g^ *p* < 0.05 vs. *Bscl2*^−/−^ ♀/♂; ^i^ *p* < 0.05 vs. *Bscl2*^−/−^ ♂; ^j^ *p* < 0.05 vs. *Bscl2*^+/Celia^ ♀/♂; ^k^ *p* < 0.05 vs. *Bscl2*^+/Celia^ ♀; ^m^ *p* < 0.05 vs. *Bscl2*^Celia/Celia^ ♀/♂ and ^p^ *p* < 0.05 vs. S.A *Bscl2*^+/Celia^.

The hepatic expression of *Fgf21*, a protective cytokine against glucolipid metabolic disorders, as well as the expression of genes related to this FGF21 complex, such as *Fgfr1*, *Klb*, and *Ppargc1a,* were assessed (Figure 6; see Supplementary Table S3).

Non-S.A. heterozygous, *Bscl2*^+/Celia^ and *Bscl2*^+/−^ animals exhibited a 60–187% significative increase in the *Fgf21* gene compared to wild-type animals. However, the increase in *Fgf21* expression was significantly higher in non-S.A. homozygous animals, both *Bscl2*^Celia/Celia^ and *Bscl2*^−/−^ (1376–1953% compared to wild-type animals). Thus, non-S.A. *Bscl2*^Celia/Celia^ mice showed an increase of 820% and 414% compared to *Bscl2*^+/Celia^ and *Bscl2*^+/−^ mice, while this increase in *Bscl2*^−/−^ animals was 1179% and 614%, respectively. Likewise, the expression of *Fgfr1* was found to be elevated in non-S.A. *Bscl2*^Celia/Celia^ animals, showing a significative increase of 58% and 70% compared to *Bscl2*^+/Celia^ and *Bscl2*^+/−^ mice.

Furthermore, an increase in *Fgf21* expression compared to wild-type and heterozygous *Bscl2*^+/Celia^ and *Bscl2*^+/−^ animals was evident in S.A. *Bscl2*^Celia/Celia^ animals, at 1451%, 867%, and 440%, respectively. Moreover, S.A. *Bscl2*^Celia/Celia^ animals showed a 3177% increase in *Fgf21* compared to S.A. *Bscl2*^+/Celia^ animals. No significant differences were found between severely affected and non-severely affected *Bscl2*^Celia/Celia^ mice. However, differences were observed among *Bscl2*^+/Celia^ mice, with severely affected animals showing a 70% decrease in *Fgf21* compared to those non-severely affected. Additionally, both *Fgfr1* and *Ppargc1a* were found to be significantly increased (approximately 1.5 to 4-fold) in S.A. *Bscl2*^Celia/Celia^ compared to wild-type and non-S.A. homozygous and heterozygous mice. There was also an increase in expression in S.A. *Bscl2*^Celia/Celia^ compared to S.A. *Bscl2*^+/Celia^, with a 285% increase in *Fgfr1* and a 156% increase in *Ppargc1a*.

No statistically significant differences in the expression of *Klb* were found between genotypes.

Sex-based analysis revealed that the *Fgf21* increase observed in heterozygous animals compared to wild-type ones was actually a difference relative to wild-type females. Additionally, *Bscl2*^−/−^ male mice exhibited a greater increase in *Fgf21* compared to *Bscl2*^Celia/Celia^ males. However, *Fgfr1* did not show significant differences between males and females. On the other hand, when considering both sexes separately, a greater increase in the expression of both *Klb* and *Ppargc1a* was observed in females compared to males.

## 3. Discussion

This study presents a detailed hepatic assessment of a humanized knock-in mouse model, which partially recapitulates PELD, previously generated by our group [20], as well as a murine model of CGL2, achieved for the first time by ubiquitous disruption of seipin via replacement of the start codon ATG with an inverted *cassette*. We confirm that the ectopic deposition of fat in the liver of the non-severely affected *Bscl2*^Celia/Celia^ and *Bscl2*^−/−^ mice leads to a pronounced hepatomegaly, with a mixed hepatic steatosis predominantly of the macrovesicular type, followed by the development of steatohepatitis and fibrosis. There is no literature on hepatic histopathology in PELD, which is limited in CGL2 and, more broadly, in any form of lipodystrophy. Some studies in CGL2 patients have reported the presence of advanced portal fibrosis [21], while others have described macrovesicular steatosis and advanced fibrosis that could progress to cirrhosis [4]. Javor et al. [3] showed a pattern of liver damage similar to that present in the current non-severely affected homozygous animals, with panacinar macrovesicular steatosis and ballooning degeneration of hepatocytes. To date, the histological analysis of the liver in lipodystrophic mice deficient in seipin has been limited to the study of isolated samples in mice at 8 weeks [22], 3 months [15,23], and 4 months [10] of age.

According to the authors of the previous studies, the SKO mice developed hepatic steatosis but not steatohepatitis or fibrosis. These findings contrast with the natural history of the disease in humans, where the development of MASH and fibrosis is a frequent and early occurrence. However, it should be noted that MASLD is a progressively evolving condition. This raises the possibility that the duration of disease progression in these earlier studies may not have been sufficient for the full spectrum of liver pathology to manifest. In support of this, Liu et al. [24] observed in 10-month-old Ad-B2^(−/−)^ mice the presence of steatosis without signs of inflammation. On the other hand, in 2018, Liao et al. [25] described, for the first time, the presence of steatohepatitis in seipin-deficient and apolipoprotein E null (Seipin^−/−^apoE^−/−^) mice at 9 months of age. This discrepancy with previous studies could be explained in two ways. Firstly, the progression time in the Liao et al. [25] study was sufficient for the development of inflammation and the full spectrum of MASLD to occur. Secondly, both in this study and the study by Liu et al. [24], we must consider whether these mice are truly comparable to the traditional SKO models. Therefore, an alternative hypothesis would be to consider that, in Liao et al. [25]’s study, two factors contribute to the development of steatohepatitis: the deficiency of seipin and, additionally, the deficiency of apoE.

It should also be noted that the non-severely affected homozygous mice in the current study only developed stage 1a fibrosis, which is mild or perisinusoidal, detectable only by Masson’s trichrome staining. Therefore, we cannot rule out the possibility that previous studies overlooked this finding, as none of them confirmed the use of this specialized staining technique [10,22,23,26]. On the other hand, in the current study, hepatic steatosis was evident from three months of age, but it only became a constant finding from six months in the homozygous animals. This raises the possibility that the progression time in the Chen et al. [26] study may have been insufficient to observe the full spectrum of liver pathology. Furthermore, McIlroy et al. [9] described that Ad-B2^(−/−)^ mice, as well as Ad-B2^(−/−)^ mice with additional ablation of hepatic *Bscl2*, did not develop hepatic steatosis [16]. Both the Ad-B2^(−/−)^ mice (studied up to 16 weeks of life) and the Ad-B2^(−/−)^ mice with additional hepatic *Bscl2* ablation (studied up to 32 weeks) showed discreet but significantly higher fat deposition and adipokine secretion capacity compared to SKO models.

According to the findings of McIlroy et al. [9], small amounts of metabolically active adipose tissue would be sufficient to prevent the development of metabolic abnormalities in SKO models. Based on this and on the fact that liver-specific SKO mice did not develop steatosis, the hepatic steatosis observed in the current study may not be directly related to the loss of a liver-specific seipin function but rather with the reduced amount of metabolically active adipose tissue [9,16], which was lower in the homozygous animals, potentially explaining the observed histopathological changes in the liver. On the other hand, Tian et al. [27], based on their observations on *Drosophila*, deficient in seipin, suggested that in tissues other than adipose, this protein promotes lipid deposition and therefore plays a tissue-specific role. The central region of seipin is proposed to control lipid homeostasis in non-adipocyte cells, being responsible for restricting lipogenesis and lipid accumulation in hepatocytes, as well as promoting adipogenesis in case of excess energy supply [13]. Therefore, the loss of seipin at the hepatic level could worsen the metabolic status in the absence or dysfunction of adipose tissue [9]. This suggests that seipin may have distinct, tissue-specific functions in regulating lipid metabolism and homeostasis, which could contribute to the complex metabolic phenotypes observed in seipin-deficient animal models and human seipinopathies.

In addition to this proposal is the observation of the FGF21 factor. In 2016, Miehle et al. [17] demonstrated for the first time a significantly higher concentration of circulating FGF21 in patients with non-HIV lipodystrophy. That same year, Dollet et al. [18] observed a marked overexpression of *Fgf21* in adipose tissue of *Bscl2*^−/−^ lipodystrophic mice at four weeks of age, which decreased at twelve weeks. In these mice, treatment with FGF21 appears to promote the maintenance of mature adipocytes by exerting an anti-stress cellular effect, improving metabolic profile. Furthermore, Softic et al. [19] noted that fat-specific insulin receptor knock-out mice (F-IRKO) exhibited increased hepatic expression of *Fgf21*. Since the accumulated fat in the livers of lipodystrophic mice is directed towards ketogenesis, the increase in this metabolic process would result from the higher hepatic expression of *Fgf21* [19].

The elevation of *Fgf21* levels observed in our homozygous *Bscl2*^Celia/Celia^ and *Bscl2*^−/−^ mice, as well as in those severely affected homozygous mice (S.A. *Bscl2*^Celia/Celia^), is consistent with previous studies and could be justified in multiple ways, although its pathogenesis remains uncertain. Upregulation of FGF21 could be considered a compensatory mechanism, yet it is insufficient to alleviate the metabolic consequences of lipodystrophy. This also applies to the increase in FGF21 in MASLD related to metabolic syndrome. Another possibility, related to circulating microRNAs (miRNAs) derived from adipose tissue, is that inhibiting gene expression acts as a posttranscriptional regulator. Thomou et al. [28], in 2017, proposed *Fgf21* as a potential target of miRNAs contained in exosomes from brown adipose tissue. They demonstrated that both hepatic expression of *Fgf21* and circulating FGF21 levels are decreased by the action of these microRNAs (primarily miR99a, miR99b, and miR100). Accordingly, the lower amount of brown adipose tissue [8] and the alteration of its function [29] in seipin-deficient mice would explain the increase in hepatic expression of *Fgf21*.

Furthermore, for FGF21 to exert its biological effects, it must interact with cell-surface FGF receptors, mainly FGFR1. Another requirement is the presence of KLB, an auxiliary protein indispensable for FGF21 to interact with FGFRs and trigger the intracellular signalling cascade that mediates biological effects of FGF21 [30,31,32,33]. However, in our study, *Klb* showed no differences in expression, while *Fgfr1* showed an increase in both S.A. *Bscl2*^Celia/Celia^ and non-S.A. *Bscl2*^Celia/Celia^ animals, but much more in the first one. This suggests a complex regulatory mechanism in which the increase in FGFR1 could be compensating for the lack of changes in KLB. Additionally, the interaction between FGF21 and PPARGC1A pathways is crucial for understanding metabolic disorders. PPARGC1A coactivates various transcription factors that control cellular energy metabolism through direct protein–protein interactions. Although PPARGC1A is expressed at relatively low levels in the adult liver under normal conditions, its expression is markedly activated during fasting and diabetes, consistent with its role as an activator of genes involved in gluconeogenesis and fatty acid oxidation [34,35]. In our study, *Ppargc1a* was found to be increased in S.A. *Bscl2*^Celia/Celia^ animals, reinforcing its role in the regulation of metabolic responses.

Beyond its hepatic actions, FGF21 also participates in immunometabolic regulation through the liver–spleen axis. β-Klotho is expressed in splenic resident macrophages and in RAW264.7 cells, where FGF21 has been shown to inhibit NF-κB activation, reduce oxi-dative stress, increase Nrf2 levels, and induce HO-1 expression [36]. Although spleen-derived macrophage responses were not evaluated in this study, these findings suggest that part of the systemic adaptation to seipin deficiency may involve FGF21-mediated modulation of inflammatory pathways beyond the liver, which warrants investigation in future work.

A limitation of the present study is that gene expression was not complemented by protein-level analysis. While the observed mRNA changes in *Fgfr1* and *Ppargc1a* were above the 2-fold threshold generally considered biologically meaningful, we acknowledge that transcriptional data alone cannot confirm functional consequences. Future studies will include Western blot and immunohistochemical analyses to validate the protein expression and spatial distribution of FGF21 signalling components in hepatic tissue.

One surprising finding was that S.A. *Bscl2*^Celia/Celia^ mice had only mild steatosis, with a quite normal liver weight. Although their food uptake was not quantified, given their severe neurological condition, the most parsimonious explanation would be that their food consumption was severely reduced. This would likely explain their lower liver weight, as well as the improvement in hepatic steatosis. In this regard, Chen et al. [11] demonstrated that prolonged fasting in SKO mice improved hepatic steatosis. This suggests that the reduced food intake in the severely affected *Bscl2*^Celia/Celia^ mice may have played a protective role against the development of more severe liver pathology. In addition to reduced food intake, the elevated expression of FGFR1 and PPARGC1A genes could also help explain, at least in part, the reduced hepatic involvement of these animals. PPARGC1A overexpression in hepatocytes enhances mitochondrial function and lipid oxidation, thereby reducing triglyceride accumulation, as observed in this study, and acting as a protective factor against steatosis [37,38].

Taken together, these findings highlight the complex interplay between seipin deficiency, adipose tissue dysfunction, and liver pathology. The observed increase in *Fgf21* levels in homozygous *Bscl2*^Celia/Celia^ and *Bscl2*^−/−^ mice, particularly in those severely affected, suggests the involvement of multiple potential mechanisms. The intricate regulatory network involving FGFR1 and KLB expression underscores the complexity of FGF21 signalling. The moderate but consistent upregulation of *Fgfr1* and *Ppargc1a* observed in mice with less hepatic involvement supports the hypothesis that these genes may participate in adaptive metabolic pathways that mitigate steatosis. Whether these findings are a consequence of lower caloric intake is something that will need to be clarified in the future. Additionally, the influence of circulating microRNAs and the upregulation of PPARGC1A offer promising avenues for further understanding and therapeutic intervention in metabolic disorders. Moreover, while murine models exhibit prominent hepatic triglyceride accumulation, the absence of cirrhosis in our study and others suggest differences in disease progression and lipotoxicity of hepatocytes between mice and humans.

In contrast, the non-severely affected homozygous animals (both knock-in and knock-out) showed a high accumulation of triglycerides in the liver, as has been reported by other authors [9,15,22,23,39]. The precise mechanisms underlying these findings are not yet well understood, but it is evident that the liver of mice may differ from that of humans in terms of lipotoxicity in hepatocytes. It is possible that the mouse liver has a greater “buffer” capacity when dealing with the spillover of fat that cannot be adequately stored, which may contribute to the differences observed in the triglyceride profiles compared to the human condition.

It is also interesting to note that, despite the long follow-up of hepatic evolution in both murine models, up to 24 months (twice as long as in most previous studies), no evidence of cirrhosis was observed. This incomplete picture of MASDL is common to all murine lipodystrophic models studied to date [40]. In contrast, some non-lipoatrophic murine models do present cirrhosis [41,42], but in these cases, the liver damage was chemically induced (by carbon tetrachloride (CCl_4_) [43], streptozotocin (STZ) [44], or diethylnitrosamine (DEN) [45]) in combination with appropriate dietary measures. The pathophysiological mechanisms employed in these models do not necessarily match the development of MASDL in humans.

Clinical evidence suggests that gender differences frequently exist in genetic forms of lipodystrophy, such as CGL2 or familial partial lipodystrophy (Dunnigan type), where female patients often exhibit a more severe physical and metabolic phenotype than male patients [46,47,48]. However, in both the current knock-in and knock-out mouse models, clear sexual dimorphism was not found. Liao et al. [25] suggested that seipin deficiency led to a similar severity of adipose loss in both female and male apoE^−/−^ mice, although females showed greater resistance to developing lipodystrophy-related metabolic consequences. These inconclusive data, inconsistent with the findings in clinical studies, were also reported by Chen. et al. [26] for their SKO murine model, where males were more affected than females. However, McIlroy et al. [49] found no major differences between genders in terms of metabolic dysfunction for the Ad-B2^(−/−)^ mice, which was also the case in the current murine models.

## 4. Materials and Methods

### 4.1. Animals and Experimental Design

All animal experiments were conducted in accordance with the European Union Directive 2010/63/EU (Directive 2010/63/EU of the European Parliament and of the Council of 22 September 2010 on the protection of animals used for scientific purposes, L 276, 20.10.2010, pp. 33–79, EUR-Lex 32010L0063, European Union, 2010) and Spanish Royal Decree 53/2013 (Royal Decree 53/2013, of February 1, establishing the basic rules applicable to the protection of animals used for experimental and other scientific purposes, including teaching, BOE-A-2013-1337, Madrid, Spain, 2013) for the protection of animals used for scientific purposes. Experimental protocols were reviewed and approved by the Ethics Committee for Animal Experimentation of the Xunta de Galicia (approval numbers 15010/17/004, 15012/2021/014, and 15012/2024/011) and by the University of Santiago de Compostela. All procedures complied with the ARRIVE 2.0 guidelines for transparent reporting of animal research [50].

#### 4.1.1. Generation of Bscl2^Celia/Celia^ and Bscl2^−/−^ Mice

For both murine models, knock-out (*Bscl2*^−/−^) and knock-in (*Bscl2*^Celia/Celia^), the ATG start codon in exon 2 of the *Bscl2* gene was replaced with a reversely positioned cassette (LoxP-Lox2272-reverse His tag-Human *BSCL2* CDS without exon7-reverse LoxP-Neo cassette-reverse Lox2272) in such a way that in the targeted allele the human transgene (3′-5′) was not transcribed before Cre mediated recombination, thus obtaining the knock-out murine model for seipin (*Bscl2*^−/−^). Upon the introduction of Cre recombinase, the inversion of the *cassette* facilitated the expression of His-tagged, aberrant human seipin, leading to the development of the knock-in murine model (*Bscl2*^Celia/Celia^) as described previously by our group [20].

The knock-in mice for the Celia seipin transgene that developed severe neurological alterations throughout their lives, as described by our group previously [20], were considered severely affected (S.A.) animals.

#### 4.1.2. Maintenance and Care of Animals

The animals were housed in ventilated racks and cages under specific pathogen-free conditions. Room temperature (22 °C ± 1 °C), humidity (55% ± 2%), and light/dark rhythm (12:12) were all controlled. All mice had ad libitum access to water and standard feed (Teklad Global 18% Protein Rodent Diet, Envigo, Inotiv, Indianapolis, IN, USA). Mice were sacrificed at ages ranging from 1.7 to 14.7 months, depending on the specific experiment; detailed information is provided in the Supplementary Tables.

#### 4.1.3. Genotyping Protocol

Isolation of genomic DNA was carried out using E.Z.N.A Tissue DNA Kit (#cat D3396-02, Omega Bio-Tek, Norcross, GA, USA). Eight specific primers were designed with the Primer3Plus software (https://www.primer3plus.com/ v.3.3.0) (last accessed on 13 December 2025)). For the seipin knock-out model genotyping strategy, three primers were combined which allowed us to distinguish between wild-type, heterozygous, and homozygous animals in one design, while for the Celia seipin knock-in model genotyping strategy, the combination of four different designs was necessary. PCR conditions and primers are available upon request. Samples were analyzed on 1% agarose gel.

### 4.2. Histology

#### 4.2.1. Tissue Processing

The tissues were fixed in 10% m/v neutral buffered formalin (cat# 05-K01022, Bio-Optica, Milano, IT, USA) for 24 h at room temperature and subsequently paraffin-embedded using a standard procedure [51]. Liver samples were sectioned at a thickness of 4 µm and stained with hematoxylin and eosin (H&E) (cat# 05-06004/L, Bio-Optica, Milano, IT, USA) or Masson’s trichrome.

For lipid deposition analysis in liver using Oil red O staining, frozen liver tissue samples were cryosectioned at a thickness of 10 µm. The sections were then fixed with 10% m/v neutral buffered formalin (cat# 05-K01022, Bio-Optica, Milano, IT, USA) for 2 h. Next, the sections were stained with Oil red O colour solution (cat# 102419, Merck-Millipore, Burlington, MA, USA). Finally, the stained sections were mounted in an aqueous mounting medium (cat# 05-1740, Bio-Optica, Milano, IT, USA).

#### 4.2.2. Imaging

The samples were observed using an Olympus BX51 microscope (Olympus, Tokyo, Japan) and captured with an Olympus DP72 digital camera. The images were acquired using the cellSens v.4.4.1 software from Olympus (Tokyo, Japan, https://www.olympus-lifescience.com/en/software/cellsens/#!cms[focus]=cmsContent6016 (last accessed on 13 December 2025)).

### 4.3. Total Non-Alcoholic Fatty Liver Disease Activity Score

The liver sections were scored blindly by two experienced pathologists using the Non-alcoholic Steatohepatitis Clinical Research Network (NASH CRN) scoring system [52]. The non-alcoholic fatty liver disease (NAFLD) activity score (NAS) ranged from 0 to 8, which was calculated as the sum of individual scores for steatosis (0–3), lobular inflammation (0–3), and hepatocyte ballooning degeneration (0–2). Moreover, the presence or absence of non-alcoholic steatohepatitis (NASH), currently known as MASLD [6], was assessed using a NAS value of ≥4 as a criterion. Non-alcoholic fatty liver (NAFL), now renamed as metabolic dysfunction-associated steatotic liver (MASL) [6], was identified if a value of ≥1 was present. Fibrosis evaluation was conducted following NASH CRN criteria, with 0 indicating the absence of fibrosis and 4 representing cirrhosis.

### 4.4. Triglyceride’s Analysis

Approximately 100 mg of liver tissue (wet weight) was homogenized in 1 mL of 2:1 (*v*/*v*) chloroform/methanol solution at 4 °C. Each liver sample was processed in duplicate. The homogenates were agitated overnight (12 rpm, 4 °C). Subsequently, 300 µL of milliQ water was added, and the samples were centrifuged (8600× *g*, 20 min, RT). The lower organic layer was collected and allowed to evaporate overnight at RT. The lipid pellets were resuspended in 900 µL (for wild-type and heterozygous samples) or 1400 µL (for homozygous samples, diluted 1:2) of chloroform [53]. Aliquots of 30 µL from each sample were evaporated (37 °C, 30–40 min). Triglyceride levels were determined using an enzymatic–colorimetric assay with the Spinreact GPO-POD kit (cat# 1001314, Spinreact, Girona, Spain). Measurements were obtained using a Multiskan™ GO Microplate Spectrophotometer (Thermo Fisher Scientific, Waltham, MA, USA).

### 4.5. RNA Isolation

Total RNA was extracted from liver using a single-step method of RNA isolation involving acid guanidinium thiocyanate–phenol–chloroform extraction and the ReliaPrepTM RNA Tissue Miniprep System kit (cat# Z6112, Promega, Fitchburg, WI, USA) [54]. RNA was reverse-transcribed using an M-MLV Reverse transcriptase kit (cat# 10338842, Invitrogen, Carlsbad, CA, USA), as previously described [55].

### 4.6. Quantitative Real-Time PCR Analysis

Specific primers and probes designed by the Universal ProbeLibrary (Roche Diagnostics, Sant Cugat del Valles, Spain) were used in a Light Cycler 2.0 (Roche Diagnostics) to determine the expression of the *Fgf21*, *Fgfr1*, *Ppargc1a*, and *Klb* genes (Table 1). Real-time qPCR analyses were performed in duplicate. Real-time PCR conditions are available upon request. Results were normalized to the *Rn18S* gene using the 2^−ΔΔ CT^ method [56].

### 4.7. Statistical Analysis

Because data distribution did not meet the assumption of normality in all variables, and sample sizes per sex and genotype were relatively small (n = 4–6), non-parametric tests were applied. Overall group differences were evaluated using the Kruskal–Wallis test, and pairwise Mann–Whitney U tests were performed only when the Kruskal–Wallis test indicated significance (*p* < 0.05). Data are presented as mean ± SD to facilitate comparison among multiple experimental groups, a common descriptive approach in small-sample biological studies. Bonferroni correction was not applied, as this conservative adjustment markedly increases the probability of type II errors (false negatives) and may obscure biologically meaningful trends. Instead, results were interpreted with caution, considering both effect size and biological plausibility. All statistical analyses were performed using IBM SPSS Statistics (release 25.0; SPSS, Chicago, IL, USA).

## 5. Conclusions

In conclusion, our study provides a detailed evaluation of hepatic pathology in mouse models of seipinopathies, shedding light on the progression of liver disease in these conditions. The observed hepatomegaly, hepatic steatosis, steatohepatitis, and fibrosis in non-S.A. homozygous, *Bscl2*^Celia/Celia^, and *Bscl2*^−/−^ mice align with clinical manifestations reported in patients with lipodystrophies, including CGL2. These results underscore the importance of comprehensive histopathological analysis to fully understand the spectrum of liver pathology associated with seipin deficiency. Our findings demonstrate that seipin loss leads to dysregulation of the FGF21 pathway, which likely reflects an adaptive response to metabolic stress rather than a primary pathogenic mechanism. The consistent upregulation of *Fgf21*, *Fgfr1*, and *Ppargc1a* in mice with limited hepatic involvement suggests their participation in protective metabolic adaptations.

Although protein-level validation remains to be performed, and the current murine models may not perfectly replicate human conditions, these results contribute valuable insight into the molecular mechanisms linking seipin dysfunction, lipid metabolism, and liver disease. They also represent significant advancements towards the development of preclinical tools for studying the pathogenesis and potential therapeutic interventions for seipinopathies, including Celia’s encephalopathy and congenital generalized lipodystrophy. Future studies integrating transcriptomic and proteomic data will further clarify the therapeutic potential of the FGF21 pathway in seipin-related disorders as continuous refinement and thorough characterization of these animal models will be pivotal in bridging the gap between murine and human manifestations of these rare genetic disorders.

## Figures and Tables

**Table 1:** Primer and probe designs for the quantitative real-time PCR. Gene name and symbol; primer sequences (forward and reverse) and probe sequences.

Gene		Primer Sequence (5′-3′)		Probe
Name	Symbol	Forward	Reverse	Sequence (5′-3′)
18S ribosomal RNA	*Rn18s*	AAACGGCTACCACATCCAAG	TACAGGGCCTCGAAAGAGTC	CGCAAATTACCCACTCCCGACCCG
Fibroblast growth factor 21	*Fgf21*	AGCATACCCCATCCCTGACT	GTACCTCTGCCGGACTTGAC	CTCCTCCA
Fibroblast growth factor receptor 1	*Fgfr1*	ATTGGAGGCTACAAGGTTCG	GAAGGCACCACAGAATCCAT	CCTGGAGC
Klotho beta	*Klb*	CGAGCCCATTGTTACCTTGT	TTTTCCAGCCCCCATATTC	CCTGGAGC
Peroxisome proliferator-activated receptor, gamma, coactivator 1 alpha	*Ppargc1a*	GAGAAGCTTGCGCAGGTAAC	TCCCATGAGGTATTGACCATC	TCCTCAGC

## Data Availability

The original contributions presented in this study are included in the article/Supplementary Material. Further inquiries can be directed to the corresponding author.

## Supplementary Materials

The following supporting information can be downloaded at: https://www.mdpi.com/article/10.3390/ijms262412037/s1.

## Abbreviations

The following abbreviations are used in this manuscript:

CGL2	Congenital generalized lipodystrophy type 2
FFA	Free fatty acids
IHBs	Intracytoplasmic hyaline bodies
MASL	Metabolic dysfunction-associated steatotic liver
MASLD	Metabolic dysfunction-associated steatotic liver disease
MASH	Metabolic dysfunction-associated steatohepatitis
NAFL	Non-alcoholic fatty liver
NAFLD	Non-alcoholic fatty liver disease
NAS	Non-alcoholic fatty liver disease activity score
NASH	Non-alcoholic steatohepatitis
PELD	Progressive encephalopathy with or without lipodystrophy
RT	Room temperature
S.A.	Severely affected
SKO	Seipin knock-out mice
