# Supplementary material for: Metabolic-Associated Steatotic Liver Disease and FGF21 Dysregulation in Seipin-Deficient and BSCL2-Associated Celia’s Encephalopathy Murine Models
Source: Int J Mol Sci. 2025 Dec 14;26(24):12037. doi: 10.3390/ijms262412037 (PMC12732938; doi:10.3390/ijms262412037)
Supplement: Supplementary file 1 [file ijms-26-12037-s001.zip › ijms-3969455-supplementary.pdf]

Supplementary information

Supplementary Table S1. Liver's weight and NAS score, fibrosis stage and NAFLD evaluation for wild type, *Bscl2*$^{+/Celia}$, *Bscl2*$^{Celia/Celia}$, *Bscl2*$^{+/-}$, *Bscl2*$^{-/-}$ and severely affected (S.A) animals. Data is presented as n (%) ± SD for liver's weight, as mean ± SD for NAS score, and as percentage (%) of animals with 1a fibrosis stage, MASL (metabolic-dysfunction associated steatotic liver) and MASH (metabolic dysfunction-associated steatohepatitis). [a] $p < 0.05$ vs wild type ♀/♂; [b] $p < 0.05$ vs wild type ♀; [c] $p < 0.05$ vs wild type ♂; [d] $p < 0.05$ vs *Bscl2*$^{+/-}$ ♀/♂; [e] $p < 0.05$ vs *Bscl2*$^{+/-}$ ♀; [f] $p < 0.05$ vs *Bscl2*$^{+/-}$ ♂; [g] $p < 0.05$ vs *Bscl2*$^{-/-}$ ♀/♂; [h] $p < 0.05$ vs *Bscl2*$^{-/-}$ ♀; [i] $p < 0.05$ vs *Bscl2*$^{-/-}$ ♂; [j] $p < 0.05$ vs *Bscl2*$^{+/Celia}$ ♀/♂; [k] $p < 0.05$ vs *Bscl2*$^{+/Celia}$ ♀; [l] $p < 0.05$ vs *Bscl2*$^{+/Celia}$ ♂; [m] $p < 0.05$ vs *Bscl2*$^{Celia/Celia}$ ♀/♂ and [p] $p < 0.05$ vs S.A. *Bscl2*$^{+/Celia}$ ♀/♂.

		N	Age (months)	Liver (%)	N	Age (months)	NAS score [0-8]	Fibrosis stage (1a)	NAFL	NASH
Wild type	♀/♂	33	9.5	3.58 ± 0.64	29	3.3	0.07 ± 0.26	0%	7%	0%
Bscl2$^{+/Celia}$	♀/♂	43	9.5	3.97 ± 0.53	27	11.4	0.37 ± 0.88	0%	22%	4%
Bscl2$^{Celia/Celia}$	♀/♂	23	9.3	16.71 ± 3.32[a,d,j]	21	9.1	3.57 ± 2.34[a,d,j,p]	38%[a,d,j]	76%[a,d,j,p]	57%[a,d,j,p]
Bscl2$^{+/-}$	♀/♂	46	9.7	3.90 ± 1.43	25	6.4	0.24 ± 0.88	0%	8%	4%
Bscl2$^{-/-}$	♀/♂	24	9.5	15.15 ± 4.09[a,d,j]	15	7.0	2.67 ± 2.41[a,d,j]	27%[a,d,j]	67%[a,d,j]	47%[a,d,j]
S.A. *Bscl2*$^{+/Celia}$	♀/♂	2	6.1	4.65 ± 0.10[a,d,g,j,m]	5	5.8	0.20 ± 0.45	0%	20%	0%
S.A. *Bscl2*$^{Celia/Celia}$	♀/♂	2	9.3	7.07 ± 0.76[a,d,g,j,m]	8	11.1	2.63 ± 1.85[a,d,j,p]	50%[a,d,j]	88%[a,d,j]	25%[a]
Wild type	♀	21	9.6	3.59 ± 0.76	16	2.7	0.06 ± 0.25	0%	6%	0%
	♂	12	9.4	3.57 ± 0.38	13	4.1	0.08 ± 0.28	0%	8%	0%
Bscl2$^{+/Celia}$	♀	26	9.5	3.95 ± 0.63[c]	11	11.3	0.18 ± 0.40	0%	18%	0%
	♂	17	9.6	4.01 ± 0.35	16	11.5	0.50 ± 1.10	0%	25%	6%
Bscl2$^{Celia/Celia}$	♀	13	9.4	16.53 ± 2.89[a,d,j]	12	11.4	4.00 ± 2.26[a,d,j]	58%[a,d,j]	83%[a,d,j]	67%[a,d,j]
	♂	10	9.1	16.93 ± 3.93[a,d,j]	9	6.0	3.00 ± 2.45[a,d,j]	11%	67%[a,f]	44%[a,f,j]
Bscl2$^{+/-}$	♀	23	9.8	4.02 ± 2.01[l]	10	5.9	0.60 ± 1.35	0%	20%	10%
	♂	23	9.6	3.78 ± 0.37	15	6.8	0.00 ± 0.00	0%	0%	0%
Bscl2$^{-/-}$	♀	10	9.5	14.62 ± 4.11[a,d,j]	6	11.7	3.67 ± 2.50[a,d,j]	33%	83%[a,d,j]	67%[a,d,j]
	♂	14	9.5	15.57 ± 4.20[a,d,l]	9	3.9	2.00 ± 2.24[e]	22%	56%[a,f]	33%[b,f]

Supplementary Table S2. Liver triglyceride analysis for wild type, *Bscl2*$^{+/Celia}$, *Bscl2*$^{Celia/Celia}$, *Bscl2*$^{+/-}$, *Bscl2*$^{-/-}$ and severely affected (S.A.) animals. Data is presented as mean ± SD. [a] $p < 0.05$ vs wild type ♀/♂; [b] $p < 0.05$ vs wild type ♀; [c] $p < 0.05$ vs wild type ♂; [d] $p < 0.05$ vs *Bscl2*$^{+/-}$ ♀/♂; [e] $p < 0.05$ vs *Bscl2*$^{+/-}$ ♀; [f] $p < 0.05$ vs *Bscl2*$^{+/-}$ ♂; [g] $p < 0.05$ vs *Bscl2*$^{-/-}$ ♀/♂; [h] $p < 0.05$ vs *Bscl2*$^{-/-}$ ♀; [i] $p < 0.05$ vs *Bscl2*$^{-/-}$ ♂; [j] $p < 0.05$ vs *Bscl2*$^{+/Celia}$ ♀/♂; [k] $p < 0.05$ vs *Bscl2*$^{+/Celia}$ ♀; [l] $p < 0.05$ vs *Bscl2*$^{+/Celia}$ ♂; [m] $p < 0.05$ vs *Bscl2*$^{Celia/Celia}$ ♀/♂; [n] $p < 0.05$ vs *Bscl2*$^{Celia/Celia}$ ♀ and [o] $p < 0.05$ vs *Bscl2*$^{Celia/Celia}$ ♂ and [q] $p < 0.05$ vs S.A. *Bscl2*$^{Celia/Celia}$ ♀/♂.

		Wild type		*Bscl2*$^{+/Celia}$		*Bscl2*$^{Celia/Celia}$		*Bscl2*$^{+/-}$		*Bscl2*$^{-/-}$		S.A. *Bscl2*$^{+/Celia}$	S.A. *Bscl2*$^{Celia/Celia}$
Liver triglycerides	Sex	♀/♂		♀/♂		♀/♂		♀/♂		♀/♂		♀/♂	♀/♂
	N	20		20		20		19		20		1	2
	Age (months)	9.5		9.6		9.3		9.5		9.5		6.8	9.3
	[mg/g liver]	7.52 ± 2.70		8.48 ± 2.34		18.06 ± 5.86[a,d,j]		9.55 ± 4.04		21.06 ± 5.65[a,d,j]		8.33	4.84 ± 3.93[g,m]
	Sex	♀	♂	♀	♂	♀	♂	♀	♂	♀	♂		
	N	10	10	10	10	10	10	9	10	10	10		
	Age (months)	9.5	9.5	9.6	9.6	9.4	9.1	9.6	9.4	9.5	9.5		
	[mg/g liver]	8.91 ± 2.83[c]	6.12 ± 1.74	9.77 ± 2.30[c]	7.20 ± 1.62[k]	17.65 ± 7.56[a,d,j]	18.46 ± 3.89[a,d,i,j]	9.01 ± 3.65	10.04 ± 4.50[c]	18.25 ± 4.37[a,d,i,j]	23.88 ± 5.53[a,d,j]		

Supplementary Table S3. Relative expression of *Ffg21, Fgfr1, Klb* and *Ppargc1a* genes in liver tissue for wild type, *Bscl2*$^{+/Celia}$, *Bscl2*$^{Celia/Celia}$, *Bscl2*$^{+/-}$, *Bscl2*$^{-/-}$ and severely affected (S.A.) mice. Data is presented as mean ± SD. Results were normalized for the *Rn18S* gene and referred to liver of wild type. $^{a}p < 0.05$ vs wild type ♀/♂; $^{b}p < 0.05$ vs wild type ♀; $^{c}p < 0.05$ vs wild type ♂; $^{d}p < 0.05$ vs *Bscl2*$^{+/-}$ ♀/♂; $^{e}p < 0.05$ vs *Bscl2*$^{+/-}$ ♀; $^{g}p < 0.05$ vs *Bscl2*$^{-/-}$ ♀/♂; $^{i}p < 0.05$ vs *Bscl2*$^{-/-}$ ♂; $^{j}p < 0.05$ vs *Bscl2*$^{+/Celia}$ ♀/♂; $^{k}p < 0.05$ vs *Bscl2*$^{+/Celia}$ ♀; $^{m}p < 0.05$ vs *Bscl2*$^{Celia/Celia}$ ♀/♂ and ^{p}p < 0.05 vs S.A *Bscl2*$^{+/Celia}$.

		Wild type		*Bscl2*$^{+/Celia}$		*Bscl2*$^{Celia/Celia}$		*Bscl2*$^{+/-}$		*Bscl2*$^{-/-}$		S.A. *Bscl2*$^{+/Celia}$	S.A. *Bscl2*$^{Celia/Celia}$
Fgf21	Sex	♀/♂		♀/♂		♀/♂		♀/♂		♀/♂		♀/♂	♀/♂
	N	28		42		18		29		15		4	10
	Age (months)	3.6		9.7		9.4		7.7		6.8		6.3	11.5
	Liver	1.49 ± 1.27		2.40 ± 2.04^{a}		22.03 ± 24.84^{a,d,j}		4.29 ± 4.98^{a}		30.64 ± 25.36^{a,d,j}		0.71 ± 0.39^{d,g,j,m}	23.15 ± 28.09^{a,j,p}
	Sex	♀	♂	♀	♂	♀	♂	♀	♂	♀	♂		
	N	15	13	18	24	8	10	12	17	6	9		
	Age (months)	3.1	4.1	9.4	9.9	14.7	5.2	6.4	8.7	10.2	4.6		
	Liver	1.33 ± 1.51	1.68 ± 0.93	2.07 ± 1.41^{b}	2.64 ± 2.41^{b}	34.78 ± 32.71^{a,d,j}	11.82 ± 8.54^{a,d,i,j}	3.51 ± 3.81^{b}	4.84 ± 5.71^{b}	32.09 ± 28.73^{a,d,j}	29.67 ± 24.64^{a,d,j}		
Fgfr1	Sex	♀/♂		♀/♂		♀/♂		♀/♂		♀/♂		♀/♂	♀/♂
	N	9		19		9		17		10		5	10
	Age (months)	3.2		5.7		8.2		2.9		4.4		5.9	12.4
	Liver	1.22 ± 0.84		1.14 ± 0.79		1.80 ± 0.97^{d,g}		1.06 ± 1.12		0.79 ± 0.61		1.14 ± 0.73	4.41 ± 2.78^{a,d,g,j,m,p}
	Sex	♀	♂	♀	♂	♀	♂	♀	♂	♀	♂		
	N	3	6	7	12	4	5	7	10	4	6		
	Age (months)	1.7	3.9	5.4	5.9	11.6	5.5	3.1	2.7	6.9	2.8		
	Liver	1.56 ± 1.29	1.05 ± 0.60	1.47 ± 0.88	0.96 ± 0.72	2.10 ± 0.95	1.56 ± 1.02	0.90 ± 0.37	1.17 ± 1.46	0.91 ± 0.81	0.71 ± 0.50		
Klb	Sex	♀/♂		♀/♂		♀/♂		♀/♂		♀/♂		♀/♂	♀/♂
	N	9		19		9		17		10		5	9
	Age (months)	3.2		5.7		8.2		2.9		4.4		5.9	12.2
	Liver	1.44 ± 1.20		2.87 ± 2.41		2.52 ± 1.51		3.50 ± 2.81		2.91 ± 2.29		1.74 ± 0.90	2.56 ± 2.39
	Sex	♀	♂	♀	♂	♀	♂	♀	♂	♀	♂		
	N	3	6	7	12	4	5	7	10	4	6		
	Age (months)	1.7	3.9	5.4	5.9	11.6	5.5	3.1	2.7	6.9	2.8		
	Liver	2.75 ± 1.11^{c}	0.78 ± 0.52	4.94 ± 2.55^{c}	1.75 ± 1.44^{e,k}	3.38 ± 1.52^{c,e}	1.84 ± 1.24^{k}	4.68 ± 3.73^{c}	2.67 ± 1.70^{c,e,k}	3.42 ± 3.12	2.57 ± 1.82^{c}		
Ppargc1a	Sex	♀/♂		♀/♂		♀/♂		♀/♂		♀/♂		♀/♂	♀/♂
	N	9		19		9		17		10		5	9
	Age (months)	3.2		5.7		8.2		2.9		4.4		5.9	12.2
	Liver	1.51 ± 1.14		1.94 ± 1.39		1.26 ± 0.79		2.59 ± 1.92		2.60 ± 2.00		2.60 ± 1.18	6.67 ± 3.62^{a,d,g,j,m}
	Sex	♀	♂	♀	♂	♀	♂	♀	♂	♀	♂		
	N	3	6	7	12	4	5	7	10	4	6		
	Age (months)	1.7	3.9	5.4	5.9	11.6	5.5	3.1	2.7	6.9	2.8		
	Liver	2.69 ± 0.50^{c}	0.91 ± 0.84	2.52 ± 0.81^{c}	1.63 ± 1.56^{e,k}	1.58 ± 0.66^{k}	1.00 ± 0.85^{e,k}	3.95 ± 2.22^{c}	1.64 ± 0.90^{e,k}	2.81 ± 1.98	2.46 ± 2.19		
